# Behavioral changes of preventive activities of influenza among children in satellite cities of a metropolitan area of Tokyo, Japan, by the COVID-19 pandemic

**DOI:** 10.1186/s12889-023-15606-x

**Published:** 2023-04-21

**Authors:** Ayako Matsuda, Kei Asayama, Taku Obara, Naoto Yagi, Takayoshi Ohkubo

**Affiliations:** 1 Center for Health Informatics policy, National Institute of Public Health, 2-3-6 Minami, Wako-Shi, 351-0197 Saitama, Japan; 2 Department of Hygiene and Public Health, Teikyo University School of Medicine, Tokyo, Japan; 3 Tohoku Institute for Management of Blood Pressure, Sendai, Japan; 4 Research Unit Hypertension and Cardiovascular Epidemiology, KU Leuven Department of Cardiovascular Sciences, University of Leuven, Leuven, Belgium; 5 Tohoku Medical Megabank Organization, Tohoku University, Sendai, Japan; 6 Warabi-Toda Medical Association, Toda, Japan

**Keywords:** Children, Influenza, Preventive measures, Behavior change, Japan, Satellite cities of metropolitan areas, Coronavirus disease 2019 season, Hand washing, Face mask wearing, Vaccination against influenza

## Abstract

**Objective:**

In children in a metropolitan area of Tokyo, Japan, behavioral change and influenza infection associated with the frequency of nonpharmaceutical interventions (NPI) was assessed from the 2018–2019 season (Preseason) and the 2020–2021 season (coronavirus disease 2019 [COVID-19] season).

**Methods:**

We conducted an exclusive survey among children attending preschool, elementary school, and junior high school in the Toda and Warabi regions, Japan, during the 2018–2019 (Preseason, distributed via mail) and 2020–2021 seasons (COVID-19 season, conducted online). The proportion of preventive activities (hand washing, face mask-wearing, and vaccination) was compared in the Preseason with that of the COVID-19 season. The multivariate logistic regression model was further applied to calculate the adjusted odds ratio (AOR) with 95% confidence intervals (CIs) for influenza infection associated with NPI frequency (hand washing and face mask wearing) in each Preseason and COVID-19 season.

**Results:**

The proportion of vaccinated children who carried out hand washing and face mask wearing was remarkably higher during the COVID-19 season (48.8%) than in the Preseason (18.2%). A significant influenza infection reduction was observed among children who washed hands and wore face masks simultaneously (AOR, 0.87; 95% CI, 0.76–0.99; *P* = 0.033).

**Conclusions:**

A strong interest and performance in the intensive measures for the prevention of influenza under the COVID-19 pandemic was demonstrated. Positive association was observed from a combination of NPI, hand washing, and face mask-wearing and influenza infection. This study’s findings could help in activities or preventive measures against influenza and other communicable diseases in children.

**Supplementary Information:**

The online version contains supplementary material available at 10.1186/s12889-023-15606-x.

## Background

The World Health Organization (WHO) reported a record-low level of influenza detection and fewer viruses were available for characterization from September 2020 to January 2021 than in previous years [1]. A similar tendency in the world can also be seen concerning an influenza epidemic in Japan [2, 3]. The coronavirus disease 2019 (COVID-19) pandemic and the ensuing related social and environmental changes apparently led to the suppression of the influenza circulation [4]. However, the influenza pandemic’s recurrence has been a significant global concern.

The cumulative COVID-19 morbidity up to January 4^th^, 2021 among children under 20 years of age in Japan was 241,662, with 45 children succumbing to the disease [5]. The Centers for Disease Control and Prevention (CDC) has developed a planning guide for vaccination clinics during the COVID-19 pandemic [6]. Additionally, the WHO and CDC have introduced information on how to prevent influenza, e.g., by washing hands and wearing a face mask [7, 8]. The WHO further recommended that wearing face masks, if to be used, must be combined with hand hygiene and other measures to prevent COVID-19’s human-to-human transmission because the use of a face mask alone is insufficient in providing an adequate protection level [9]. The CDC’s Science Brief, based on experimental and epidemiological evidence, supported the use of community face masking to reduce the spread of the SARS-CoV-2 virus among children [10]. Xiao et al. reviewed the evidence based on the effectiveness of nonpharmaceutical personal protective measures and environmental hygiene measures [11]. They stated that although the mechanistic studies support the potential effect of hand hygiene or face masks, evidence from randomized controlled trials (RCTs) of these measures from 2006–2014 for countries other than Japan did not support a substantial preventive effect on laboratory-confirmed influenza transmission [11]. A previous study conducted in 2012 suggested that the use of face masks in public was typically associated with better personal hygiene practices and health behaviors among Japanese adults [12]. However, the state of preventive activities and interests during the COVID-19 season in Japan is unclear.

An exclusive survey study on children in two satellite cities of Tokyo, Japan, during the 2018–2019 (Preseason) and 2020–2021 seasons (COVID-19 season) was conducted as significant attention was paid to behavioral changes as a consequence of non-pharmaceutical interventions (NPI) during the pandemic. The study aims to examine the prevalence and clinical efficacy of washing hands and wearing face masks in addition to vaccination against influenza by comparing the preventive effects of these measures before and during the COVID-19 season. The association between NPI frequency during pre-season and the COVID-19 season, behavioral changes and influenza infections were assessed by controlling for potential confounding factors.

## Methods

### Study procedure

Study area and study design details have been published previously [13, 14]. Briefly, we have conducted an annual survey among children attending preschool, elementary school, and junior high school, and their parents in the Toda and Warabi cities, Saitama, Japan. In the study area, the total number of children aged ≤ 15 years was 28,029 according to the 2020 census [14]. The survey in the 2019–2020 season was cancelled because this was the initial year of the COVID-19 pandemic. The Preseason survey was conducted in June 2019 by mail survey [13, 14] and the survey in the COVID-19 season was conducted from May to July 2021 by an online research support system. The class schoolteacher gave information about the online survey in June 2021; a unique identifier (ID) was printed on each information letter to eliminate duplicate answers, but was not linked to each respondent. The children’s parents scanned the two-dimensional code (QR code) provided on the letter, reported the ID, and answered the subsequent questions online by themselves. The website used to support this research was developed in association with QLife Inc. (Tokyo, Japan).

### Statistical analysis

In this analysis, each influenza season was defined as starting in October of the current year and ending in March of the succeeding year. For instance, the COVID-19 season (2020–2021) began in October 2020 and ended in March 2021.

In the characteristics of the children and the need for influenza vaccination, comparisons between Preseason and the COVID-19 seasons were compared using the $\upchi$^2^ test.

The $\upchi$^2^ test was used to compare the preventive activities (nothing; hand washing only; face mask wearing only; vaccination against influenza only; hand washing and face mask wearing; hand washing and vaccination; face mask wearing and vaccination against influenza; and hand washing, face mask wearing, and vaccination against influenza) in the Preseason with those during the COVID-19 season. The multivariate logistic regression model was used to calculate the adjusted odds ratio (AOR) with 95% confidence intervals (CIs) for the influenza infection associated with the NPI (hand washing only, face mask wearing only, hand washing and face mask wearing) frequency in each Preseason and COVID-19 season. This study controlled the potentially confounding roles of school (preschool, elementary school, and junior high school), gender (male/female), siblings (yes/no), underlying diseases (yes/no), and vaccination (yes/no) in the model. For missing values of washing hands (*n* = 9), wearing face masks (*n* = 72), and need for vaccination (*n* = 74) in Preseason, the missing variable values were excluded from the analysis. Additionally, a sensitivity analysis that estimated adjusted odds ratios for influenza infection, with vaccination against influenza being considered as a confounding factor, was also performed. The assessed categories included washing hands only, wearing face mask only, vaccination against influenza only, washing hands + wearing face mask, washing hands + vaccination against influenza, wearing face mask + vaccination against influenza, and washing hands + wearing face mask + vaccination against influenza. Children practicing none of the above preventive measures were included as the reference category. The goodness of fit of the model (*P* > 0.05) was checked using the Hosmer–Lemeshow test.

Statistical significance was considered a *P* value of < 0.05. The Stata version 16.0 (Stata Corp., College Station, TX, USA) and SAS (Statistical Analysis Software 9.4, SAS Institute Inc., Cary, NC, USA) were used to analyze data.

## Results

**Fig. 1:**
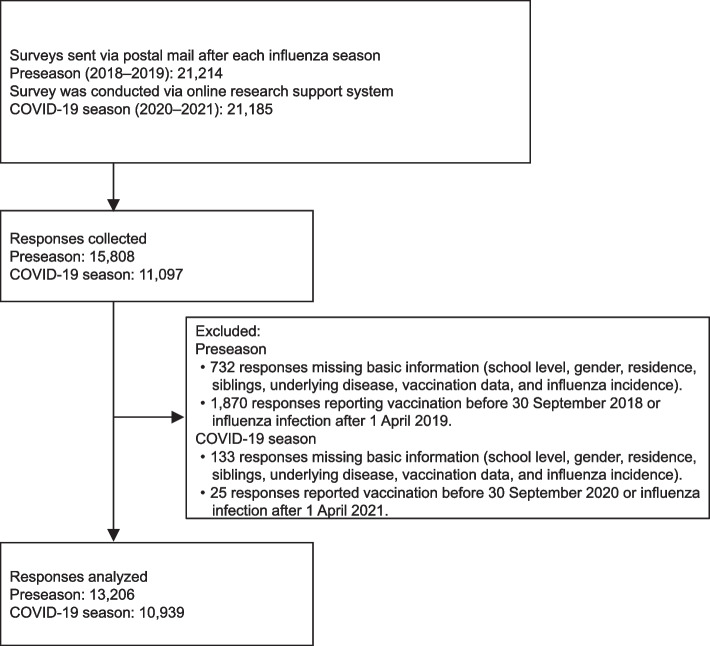
Study population selection

The mail survey in the Preseason was distributed among 21,214 parents of children attending preschool, elementary school, or junior high, and the response rate was approximately 70.8% (*n* = 15,808). A total of 11,097 responses in the COVID-19 season were collected using the survey conducted via the online research support system for 21,185 parents of children (response rate, 52.4%). Respondents who did not answer the basic information (*n* = 732 and 133 in the Preseason and COVID-19 season, respectively) and had influenza vaccination before September 30 or the prevalence of children with influenza after April 1 for each season (*n* = 1,870 and 25) were excluded [15]. This analysis consisted of 13,206 respondents in the Preseason and 10,939 respondents during the COVID-19 season (Fig. 1).

The characteristics of the children in this study between the Preseason and COVID-19 season are displayed in Table 1. The boy-girl ratio was 50:50, irrespective of the catchment seasons (*P* = 0.770). Most children had siblings, which was also commonly observed with no significant difference between the two seasons (*P* = 0.443). In contrast, the prevalence in children with underlying medical problems was higher in the Preseason (7.8% vs. 4.6%; *P* < 0.001). The proportion of children who frequently washed their hands (88.0%) and wore face masks (83.3%) during the COVID-19 season increased compared to that in the Preseason (70.7% and 41.6%, respectively). Furthermore, the parents of the children’s proportions who agreed to the influenza vaccination necessity (59.4%) and received vaccination (60.9%) in the COVID-19 season were more than those during the Preseason (52.0% and 46.0%, respectively).

**Table 1:** Characteristics of children in the cities of Toda and Warabi, Japan

**Variables**		**Preseason**	**COVID-19 Season**	
		**(*n* = 13,206)**	**(*n* = 10,939)**	***P* value**
Catchment season		2018–2019	2020–2021	
Girl sex, n (%)		6610 (50.0)	5496 (50.2)	0.770
Siblings, n (%)		10,389 (78.7)	8561 (78.3)	0.443
Underlying medical problem, n (%)		1031 (7.8)	504 (4.6)	< 0.001
School, n (%)	Preschool (0–6 years)	3310 (25.1)	2399 (21.9)	< 0.001
	Elementary school (7–12 years)	7652 (57.9)	6414 (58.6)	
	Junior high school (13–15 years)	2244 (17.0)	2126 (19.5)	
Washing hands, n (%)	Not frequently	3866(29.3)	1313(12.0)	< 0.001
	Frequently	9331 (70.7)	9626 (88.0)	
	Missing information	9	0	
Face mask wearing, n (%)	Not frequently	7668 (58.4)	1823 (16.7)	< 0.001
	﻿Frequently	5466 (41.6)	9116 (83.3)	
	Missing information	72	0	
Need of vaccination, n (%)	Agree	6829 (52.0)	6494 (59.4)	< 0.001
	Disagree/not sure	6303 (48.0)	4445(40.6)	
	Missing information	74	0	
Vaccination, n (%)	Yes	6071 (46.0)	6659 (60.9)	< 0.001
	No	7135 (54.0)	4280 (39.1)	

Data from Preseason and COVID-19 seasons were compared using the $\upchi$^2^ test

### Preventive measures

The preventive activities’ implementation status among children between the Preseason and COVID-19 season is displayed in Table 2. In all preventive activities (nothing; hand washing only; face mask wearing only; vaccination against influenza only; hand washing and face mask wearing; hand washing and vaccination against influenza; face mask wearing and vaccination against influenza; and hand washing, face mask wearing, and vaccination against influenza), they were significantly different compared from the Preseason and COVID-19 season. Specifically, the proportion of vaccinated children who carried out hand washing and face mask wearing was remarkably higher in the COVID-19 season (48.8%) than in the Preseason (18.2%).

**Table 2:** Preventive measures and their combinations

**Measures**	**Answer**	**Preseason**	**COVID-19 Season**	
		(*n* = 13,125)*	**(*n* = 10,939)**	***P* value**
Not taking any preventive measure		2067 (15.7)	368 (3.4)	< 0.001
Washing hands Only, n (%)	Frequently	2193 (16.7)	401 (3.7)	< 0.001
Wearing face mask Only, n (%)	Frequently	523 (4.0)	380 (3.5)	0.038
Vaccination Only, n (%)	Yes	994 (7.6)	304 (2.8)	< 0.001
Washing hands + Wearing face mask, n (%)	Frequently	2300 (17.5)	3131 (28.6)	< 0.001
Washing hands + Vaccination, n (%)	Frequently or yes	2411 (18.4)	750 (6.8)	< 0.001
Wearing face mask + Vaccination, n (%)	Frequently or yes	250 (1.9)	261 (2.4)	0.010
Washing hands + Wearing face mask + Vaccination, n (%)	Frequently or yes	2387 (18.2)	5344 (48.8)	< 0.001

Preseason and COVID-19 season denotes the 2018–2019 season and 2020–2021 season, respectively. Data from Preseason and COVID-19 seasons were compared by the $\upchi$^2^ test

^*^Participants with missing values (*n* = 81) were excluded from the analysis

### NPI and influenza infection association among children

**Fig. 2:**
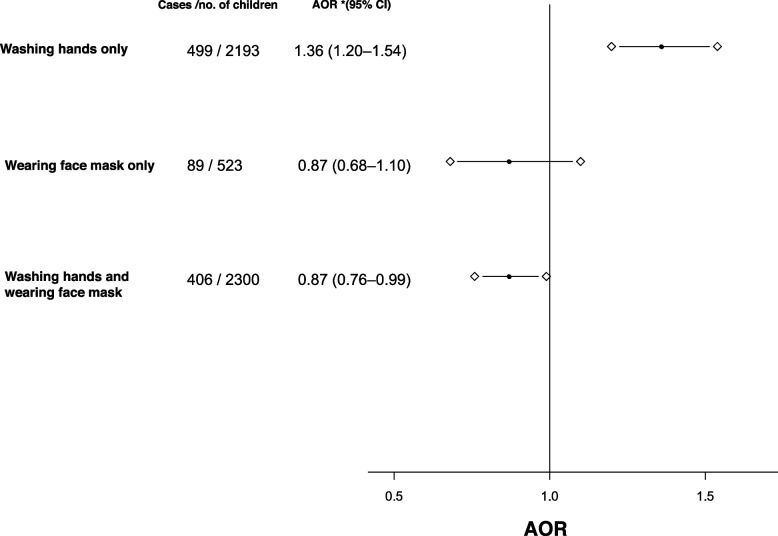
Adjusted odds ratios (AORs) for influenza infection in nonpharmaceutical interventions (NPI; hand washing only, face mask wearing only, hand washing and face mask wearing) during Preseason. For missing values of washing hands (*n* = 9) and face mask wearing (*n* = 72), the missing variable values from the analysis were excluded. The goodness of fit of all models was determined using the Hosmer–Lemeshow test (*P* > 0.05). *Odds ratios with 95% confidence intervals (CI) were adjusted for school, gender, sibling, underlying disease, and vaccination

The association between the NPI and influenza infection in the Preseason is shown in Fig. 2. The association in the COVID-19 season could not be evaluated because there were only 20/10,939 children infected with influenza.

The preventive effect of each measure alone was vulnerable; among children who washed hands only and wore face masks only, the AOR for frequently was 1.36 (95% CI, 1.20–1.54; *P* < 0.001), 0.87 (95% CI, 0.68–1.10; *P* = 0.256), respectively. In contrast a significant reduction of influenza infection was observed (AOR, 0.87; 95% CI, 0.76–0.99; *P* = 0.033) among children who washed hands and wore face masks simultaneously. Regarding the fit of the model, all the multivariate logistic regression models showed a statistically good fit (Hosmer–Lemeshow test, *P* > 0.05).

The supplementary table shows the adjusted odds ratios for influenza infection during the pre-season, evaluated using the sensitivity analysis. The AORs following hand washing only, face mask wearing only, and hand washing and face mask wearing were considered to have similar tendencies in the AORs following the NPI frequency as the reference.

## Discussion

In the Preseason, Japanese children have already been washing hands and wearing face masks at a high rate (70.7% and 41.6%, respectively). The previous study that included all children attending elementary schools in Matsumoto City, Japan, in 2005 found that NPIs included wearing face masks (*n* = 5474 children; 52.0%) and hand washing (*n* = 8322 children; 79.1%) [16]. A similar tendency was also observed in the current study during the pre-season. The preventive activities for children were even more popular, and more children received influenza vaccination during the COVID-19 season. Approximately half of the children carried out all of the hand washing and face mask wearing frequently, and was vaccinated during the COVID-19 season. As for the NPI and influenza infection association among children in the Preseason, face mask wearing combined with hand washing significantly reduced the risk of influenza infection. In contrast, hand washing alone was associated with a greater risk of influenza infection.

Multiple factors have likely contributed to the marked decline in the influenza virus’ circulation during the COVID-19 season, including the implementation of community mitigation measures to control the SARS-CoV-2 transmission, behavioral changes in response to the pandemic, and influenza vaccination [17–19]. Vaccination is the primary strategy for the prevention and control of influenza [20], especially for children [21], and hygiene methods such as hand washing, face masks, and quarantine are reported to be effective [22, 23]. The Preseason survey was completed in March 2019 and was not affected by the COVID-19 pandemic. In contrast, the 2020–2021 season survey was greatly influenced by COVID-19. Therefore, they should be carefully interpreted because of changes in individual-level behavior or public health response measures according to the COVID-19 pandemic. Nevertheless, this survey emphasized and demonstrated the different states of preventive activities and interests of influenza in children between the Preseason and COVID-19 seasons, which may encourage the promotion of preventive activities of influenza in the future.

A systematic NPI review for pandemic influenza [11] provided negative conclusions, as the evidence from the RCTs suggested that both hand hygiene and face mask interventions do not have a substantial effect on influenza transmission. A previous observational study of all elementary schoolchildren in Japan showed that hand washing was associated with a greater likelihood of developing seasonal influenza [16]. However, the likelihood of developing seasonal influenza was decreased by face mask wearing [16]. This study’s findings were consistent with this study of the reverse direction [16]. Furthermore, another web-based survey has reported that wearing a face mask in public was associated with other positive personal hygiene practices and health behaviors among Japanese adults [12]. This study provides the effectiveness of wearing face mask combined with hand washing on influenza for children. The prospect of an influenza epidemic after the COVID-19 pandemic has subsided and remains unknown. Health promotion efforts should be aimed at increasing preventive awareness of influenza infection to better protect the general public against future influenza epidemics [24]. As Zipfel et al. stated, we must listen to the many lessons learned and relearned about influenza dynamics and control over the pandemic to prepare for future outbreaks [25]. A continuous survey of the infections would be required. At the very least, the findings from our influenza survey for children are helpful in future influenza and other infectious disease measures.

### Strengths and limitations

Several limitations associated with the present study warrant mention. First, we used a questionnaire that did not request detailed medical information; therefore, the answers regarding influenza infection might not be accurate. However, we emphasize that the influenza antigen rapid test is widely available in Japan [14]. We are confident about the influenza diagnosis, even though it is based on the questionnaire. Second, the COVID-19 season survey was conducted through an online research support system, which might have caused bias in comparison with the survey in the Preseason. However, this survey has been carried out with similar structured questions, with schoolteachers verifying the flow of responses. Furthermore, this study was able to avoid missing data during the COVID-19 season survey by not allowing missing answers in the online system. Third, a definitive conclusion cannot be made on the NPI’s effectiveness on influenza because the present study was not an RCT. However, it is difficult to provide generalizable results in an RCT given the limited population and it might not always reflect the natural setting of an influenza epidemic [23]. This study’s results demonstrated high generalizability considering that the population was collected in an exclusive manner in satellite cities of a metropolitan area of Tokyo, and the obtained results were multivariable adjusted. Fourthly, washing hands and wearing a face mask were not evaluated quantitatively and only a qualitative assessment using scores ranging from 1 to 3 (1: never; 2: somewhat; 3: frequently) was carried out. Therefore, the estimation of the effectiveness of washing hands and wearing a face mask may have been imprecise**.** Finally, the external validity of the questionnaire items has not been examined. However, its high internal validity and ability to assess the prevalence and effects of influenza vaccinations allows us to provide some notable findings [13, 14].

As a strength of our study, we successfully demonstrated a strong interest and performance in the intensive measures for the prevention of influenza under the COVID-19 pandemic in two satellite cities near Tokyo, based on survey data from the same region. After adjusting for confounding factors, the result of the current study showed a positive association between the combination of NPI, hand washing, face mask wearing, and influenza infection among Japanese children. Sawakami T suggested that the behavioral changes adopted to prevent COVID-19 transmission in Japan could serve as a valuable reference for reducing the spread of seasonal influenza in the future [26].

## Conclusion

The findings of the current study may be used to support recommendations for preventing common influenza and COVID-19 among children. The recommendations for preventing common influenza and COVID-19 among children provided in the current study are particularly relevant to those living in metropolitan areas, similar to those in our study, but can also be applied to other children populations. This study’s findings could aid in creating activities or preventive measures against influenza, and perhaps against other communicable diseases, in children.

## Supplementary Information

**Additional file 1:** **Supplementary Table.** Adjusted odds ratios for influenza infection during the Preseason (the 2018–2019 season).

## Data Availability

The datasets generated and/or analyzed in the current study are available from the corresponding author upon reasonable request.

## Abbreviations

AOR: Adjusted odds ratio
CDC: Centers for disease control and prevention
CI: Confidence intervals
COVID-19: Coronavirus disease 2019
ID: Identifier
NPI: Nonpharmaceutical interventions
RCT: Randomized controlled trials
WHO: World health organization
