# Supplementary material for: Behavioral changes of preventive activities of influenza among children in satellite cities of a metropolitan area of Tokyo, Japan, by the COVID-19 pandemic
Source: BMC Public Health. 2023 Apr 21;23:727. doi: 10.1186/s12889-023-15606-x (PMC10119014; doi:10.1186/s12889-023-15606-x)
Supplement: Supplementary file 1 — Additional file 1: Supplementary Table. Adjusted odds ratios for influenza infection during the Preseason (the 2018–2019 season). [file 12889_2023_15606_MOESM1_ESM.docx]

**Supplementary Table. Adjusted odds ratios for influenza infection during the Preseason (the 2018–2019 season).**

| **Measures** | AOR (95% CI)* |
| --- | --- |
| Not taking any preventive measure | 1 |
| Washing hands Only | 1.35 (1.16-1.58) |
| Wearing face mask Only | 0.96 (0.74-1.24) |
| Vaccination Only | 1.03 (0.84-1.25) |
| Washing hands + Wearing face mask | 1.00 (0.85-1.17) |
| Washing hands + Vaccination | 1.14 (0.98-1.33) |
| Wearing face mask + Vaccination | 1.19 (0.85-1.63) |
| Washing hands + Wearing face mask + Vaccination | 1.04 (0.89-1.21) |

Children practicing none of the preventive measures are considered as the reference category. Missing values for washing hands (*n* = 9) and face mask wearing (*n* = 72) were excluded from the analysis. The goodness of fit for all models was determined using the Hosmer–Lemeshow test (*p-value* > 0.05).

*Odds ratios with 95% confidence intervals (CI) were adjusted for school, gender, sibling, and underlying disease.
